# RadCLARE: an automated clinical language engine for detecting semantic errors in radiology reports

**DOI:** 10.1186/s41747-025-00659-x

**Published:** 2025-12-22

**Authors:** Feng Pan, Jie Lou, Yusheng Guo, Wang Du, Zhonghua Wang, Qianqian Fan, Hao Wang, Chuansheng Zheng, Lian Yang

**Affiliations:** 1 Department of Radiology, Union Hospital, Tongji Medical College, Huazhong University of Science and Technology, Wuhan, China; 2 Hubei Provincial Clinical Research Center for Precision Radiology & Interventional Medicine, Wuhan, China; 3 Hubei Key Laboratory of Molecular Imaging, Wuhan, China; 4 WanLiCloud Healthcare IT Co., Ltd., Beijing, China; 5 Information and Data Center, Union Hospital, Tongji Medical College, Huazhong University of Science and Technology, Wuhan, China

**Keywords:** Artificial intelligence, China, Language, Medical errors, Radiology

## Abstract

**Background:**

Errors in radiology reports can result in inappropriate/harmful decisions. We investigated whether large language models can reduce the error rate.

**Materials and methods:**

We developed the radiology-specific clinical language anomaly recognition engine (RadCLARE) network, an automated engine based on the bidirectional encoder representations from transformers (BERT)-base model, designed to detect semantic errors in Chinese radiology reports and trained using 1.4 million reports, including 615,920 digital radiography, 560,310 computed tomography reports, and 223,480 magnetic resonance reports. One thousand reports were randomly selected for expert manual annotation. Inter-reader agreement for error detection and classification was assessed using Cohen κ and Gwet AC1. The RadCLARE’s detection was compared against the expert references. Changes in error rates before (baseline test dataset, BTD) and after (experimental test dataset, ETD) RadCLARE implementation were analyzed. Finally, radiologists were invited to complete questionnaires to evaluate satisfaction and rate the system across five dimensions.

**Results:**

Among the 1,000 reports, a total of 506 errors were identified as the reference standard. Inter-reader agreement was substantial for error detection (κ = 0.77) and excellent for error classification (Gwet AC1 = 0.94). RadCLARE successfully detected 437/506 errors, with 87.3% accuracy, 88.3% precision, 86.4% recall, and 87.4% F1-score. The BTD comprised 571,264 reports, the ETD 873,030 reports. After RadCLARE implementation, the semantic error rate dropped significantly compared to the BTD (error rate, 0.85% [7408/873,030] *versus* 4.19% [23,909/571,264]; *p* < 0.001). The questionnaire results showed that 95.7% (44/46) of radiologists were satisfied with RadCLARE.

**Conclusion:**

RadCLARE showed the capability for automatic detection of semantic errors in radiology reports.

**Relevance statement:**

RadCLARE demonstrated high performance in detecting semantic errors in radiology reports. Future studies should aim to extend their applicability across multiple languages and institutions.

**Key Points:**

- We developed the RadCLARE network, a BERT-based engine for detecting semantic errors in Chinese radiology reports.
- With the aid of RadCLARE, the semantic error rate in radiology reports dropped significantly from 4.19% to 0.85%.
- The large majority (96%) of radiologists who participated in the test were satisfied with the RadCLARE and felt that it reduced stress.

**Graphical Abstract:**

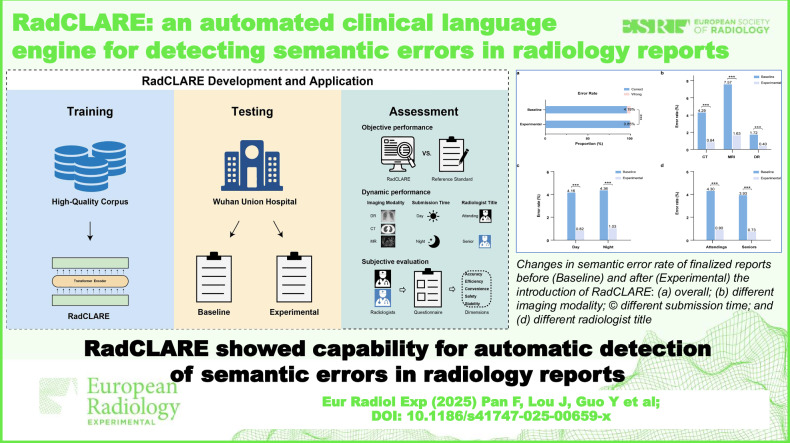

## Background

In most radiology departments, preliminary reports are initially drafted by residents, who interpret medical images and provide their findings. Then, these preliminary reports are reviewed by radiologists certified by the departmental review board, which could increase the accuracy and consistency of radiology reports [1]. Despite these quality assurance mechanisms, practical challenges remain, such as increasing workload and fatigue [2, 3]. In the United States, the error rate in radiology reports is estimated to be as high as 10–15%, with an average daily error rate of 3–5% [4]. Common writing errors include spelling mistakes, side confusion, and incorrect measurement units, all of which can result in serious consequences, such as unnecessary or inappropriate treatment [5]. Therefore, reducing errors in radiology reports is a critical issue that must be addressed in clinical practice.

The advent of artificial intelligence (AI) offers a promising solution to the persistent challenges of radiology report errors. Among various AI-driven approaches, large language models (LLMs) have demonstrated remarkable capabilities in the field of radiology, including error detection, context understanding, disease detection, and automated text refinement [6–11]. LLMs are particularly well-suited for radiology reporting because they can process large amounts of text data, recognize structural patterns in reports, and identify semantic inconsistencies, linguistic ambiguities, and factual inaccuracies that might be overlooked by human reviewers [7, 12]. Moreover, LLMs can assist in standardizing report terminology, enhancing report completeness, and reducing interobserver variability, ultimately improving diagnostic accuracy and communication with referring radiologists [13].

In this study, we developed RadCLARE (radiology-specific clinical language anomaly recognition engine), a deep learning model based on the bidirectional encoder representations from transformers (BERT)-base, specifically designed to automatically detect a variety of errors in Chinese radiology reports. Fine-tuned on a high-quality medical imaging corpus, RadCLARE enables real-time semantic error detection (*e.g*., gender mismatches, side confusion, etc.) with an average processing time of less than five seconds, making it highly suitable for fast-paced clinical environments. Unlike traditional rule-based systems, RadCLARE adopts a dual-detection approach that combines LLM contextual understanding with a knowledge-rule-based mechanism, enhancing its ability to identify and validate errors. The system is designed to enhance radiologists’ reporting by reducing semantic errors, standardizing terminology, and improving report consistency and completeness. To evaluate its real-world performance, we compared retrospective data collected prior to RadCLARE implementation with prospective data gathered after its deployment. This allowed us to assess the impact of RadCLARE on radiologists’ writing performance in routine clinical practice.

## Materials and methods

This study was approved by the Ethics Committee of Wuhan Union Hospital (No. 2024-0845), and informed consent was waived by the Institutional Review Board. This study implemented stringent data protection measures, ensuring that all data were anonymized to protect privacy.

### Definitions of errors

To effectively train the model, we first established clear definitions of semantic errors to be used as labels. Errors were categorized into seven types consistent with previous research [5, 14]:

- spelling mistakes;
- side confusion;
- incorrect measurement units;
- gender errors;
- age errors;
- mismatched imaging modalities;
- other.

The definitions and examples of these errors are listed in Supplementary Table S1.

### Training dataset

To effectively train the RadCLARE network, we prepared a well-annotated, large-scale training dataset. The dataset consisted of approximately 1.4 million finalized radiology reports, retrospectively collected between September 1, 2010, and December 1, 2020. These included:

- 615,920 digital radiography (DR) reports;
- 560,310 computed tomography (CT) reports;
- 223,480 magnetic resonance imaging (MRI) reports.

All reports underwent rigorous cross-review and proofreading by a panel of 16 attending radiologists to ensure accuracy and eliminate errors. Additionally, the attending radiologists manually annotated 11 key entities in the reports, including:

- orientations: *e.g*., “左” (left), “右” (right), “上” (upper), etc.;
- anatomical locations: *e.g*., “心脏” (heart), “肺部” (lung), 肺纹理 (lung texture), etc.;
- qualitative terms: *e.g*., “可见” (visible), “弥漫” (diffuse), “恶性” (malignant), etc.;
- size-related terms: *e.g*., “大” (big), “小” (small), “中等” (moderate), etc.;
- measurement units: *e.g*., “cm”, “mm”, “HU”, etc.;
- positive signs: *e.g*., “阴影” (shadow), “结节” (nodule), 肿物 (mass), etc.;
- negative signs: *e.g*., “通畅” (patent), “清晰” (clear), “阴性” (negative), etc.;
- diagnosis: *e.g*., “肺炎” (pneumonia), “感染” (infection), “炎症” (inflammation), etc.;
- gender-related terms: *e.g*., “前列腺” (prostate), “男” (male), “子宫” (uterus), etc.;
- device-related terms: *e.g*., “X线” (x-ray), “磁共振” (MRI), “密度” (density), etc.;
- age-related terms: *e.g*., “腺样体” (adenoids), “前囟” (anterior fontanel), “松果体钙化” (pineal calculus), etc.;

Each entity could belong to multiple categories. For example, “前列腺” (prostate) was classified under both “anatomical location” and “gender-related terms”.

### High-quality corpus used for error generation

To simulate erroneous reports, we employed a systematic perturbation approach. First, we constructed a high-quality corpus by integrating the following resources: (a) commonly used phrases from imaging reports (Supplementary Table S2); (b) Radiological Society of North America–RSNA radiology lexicon (RadLex) (https://www.rsna.org/practice-tools/data-tools-and-standards/radlex-radiology-lexicon); (c) homophone and near-homophone dictionaries (Supplementary Table S3); and (d) a general medical thesaurus (https://www.meddra.org/). Then, entities or words within a sentence were randomly replaced with plausible alternatives, or words were randomly omitted or inserted, to generate the incorrect reports. Examples of these generated errors are provided in Supplementary Table S4.

### RadCLARE architecture and training process

**Fig. 1:**
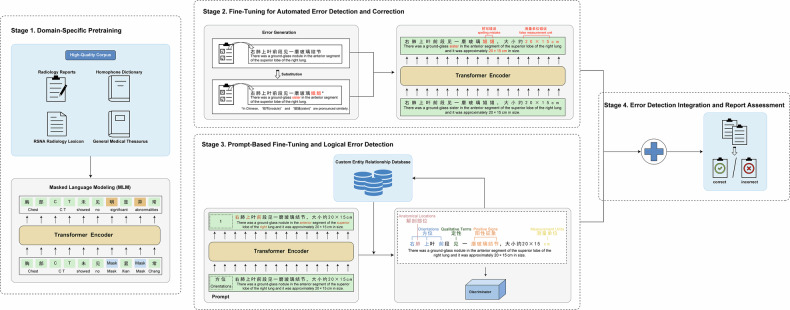
RadCLARE training workflow. RadCLARE, Radiology-specific clinical language anomaly recognition engine

The BERT-base model served as the backbone of RadCLARE. BERT is a transformer-based model that uses self-attention mechanisms to capture bidirectional contextual relationships within text [15]. The BERT-base configuration comprises 12 transformer layers, each with 12 attention heads, totaling 110 million parameters. Its bidirectional nature enables richer contextual embeddings, making it well-suited for processing radiology texts. To adapt BERT for this domain, the model was trained through four sequential stages (Fig. 1).

Stage 1—domain-specific pretraining: RadCLARE was initialized with pre-trained BERT-base weights and further trained using a masked language modeling–MLM objective on the collected radiology reports to adapt to domain-specific vocabulary, syntax, and semantics [16, 17].

Stage 2—fine-tuning for automated error detection and correction: using paired radiology reports (correct *versus* incorrect), RadCLARE was optimized to detect and categorize writing errors. Different types of errors were artificially generated via entity substitution using the high-quality corpus as mentioned earlier. Approximately 15% of the tokens were randomly masked during training. The model was then trained to predict the original words based on the context. Through this process, the model developed the ability to identify and correct errors within a contextual understanding framework. For example, given an erroneous phrase like “左肺商业” (“left lung business”), the model can infer the correct medical term “上叶” (“upper lobe”) based on the surrounding context “左肺” (“left lung”).

Stage 3—prompt-based fine-tuning and logical error detection: key entities as mentioned above (*e.g*., anatomical locations, orientations, etc.) were extracted using prompt-based fine-tuning. Afterward, these entities were compiled into an entity relationship database encoding valid and invalid relationships based on the classifications of correct and incorrect reports. Supplementary Table S5 provides representative examples from the custom entity relationship database. If a relationship was judged invalid, we classified it into one of the predefined seven error types based on the entity types. In the test stage, logical consistency checks were performed based on the compiled relationship rules to ensure contextual accuracy.

Stage 4—error detection integration and report assessment: outputs from Stage 2 (error detection and correction) and Stage 3 (logical verification) were integrated. The combined system provided final report assessments with detected errors and suggested corrections.

### Test datasets and deployment workflow

The test dataset was collected from the Department of Radiology, Union Hospital, Tongji Medical College, Huazhong University of Science and Technology. Data collection was conducted in two phases:

1. Baseline test dataset (BTD): finalized reports were retrospectively collected from July 1, 2022, to June 30, 2023, representing conventional reporting practices before the implementation of RadCLARE.
2. Experimental test dataset (ETD): finalized reports were prospectively collected from July 1, 2023, to October 31, 2024, during which RadCLARE was integrated into the radiology reporting workflow.

During the deployment phase, RadCLARE was integrated into the reporting system via an Application Programming Interface-based service model. The system does not interfere with the normal workflow of radiologists during the report-writing process. Once a report has been submitted, RadCLARE automatically initiates error detection and completes quality control analysis within a few seconds. The results of the detection process are fed back to the front-end interface in a structured JavaScript Object Notation format. If errors are identified, the system promptly alerts the relevant doctors via a pop-up window, ensuring that errors can be swiftly identified and corrected. A diagram of the RadCLARE user interface is provided in Supplementary Fig. S1.

### Assessments of the model performance

**Fig. 2:**
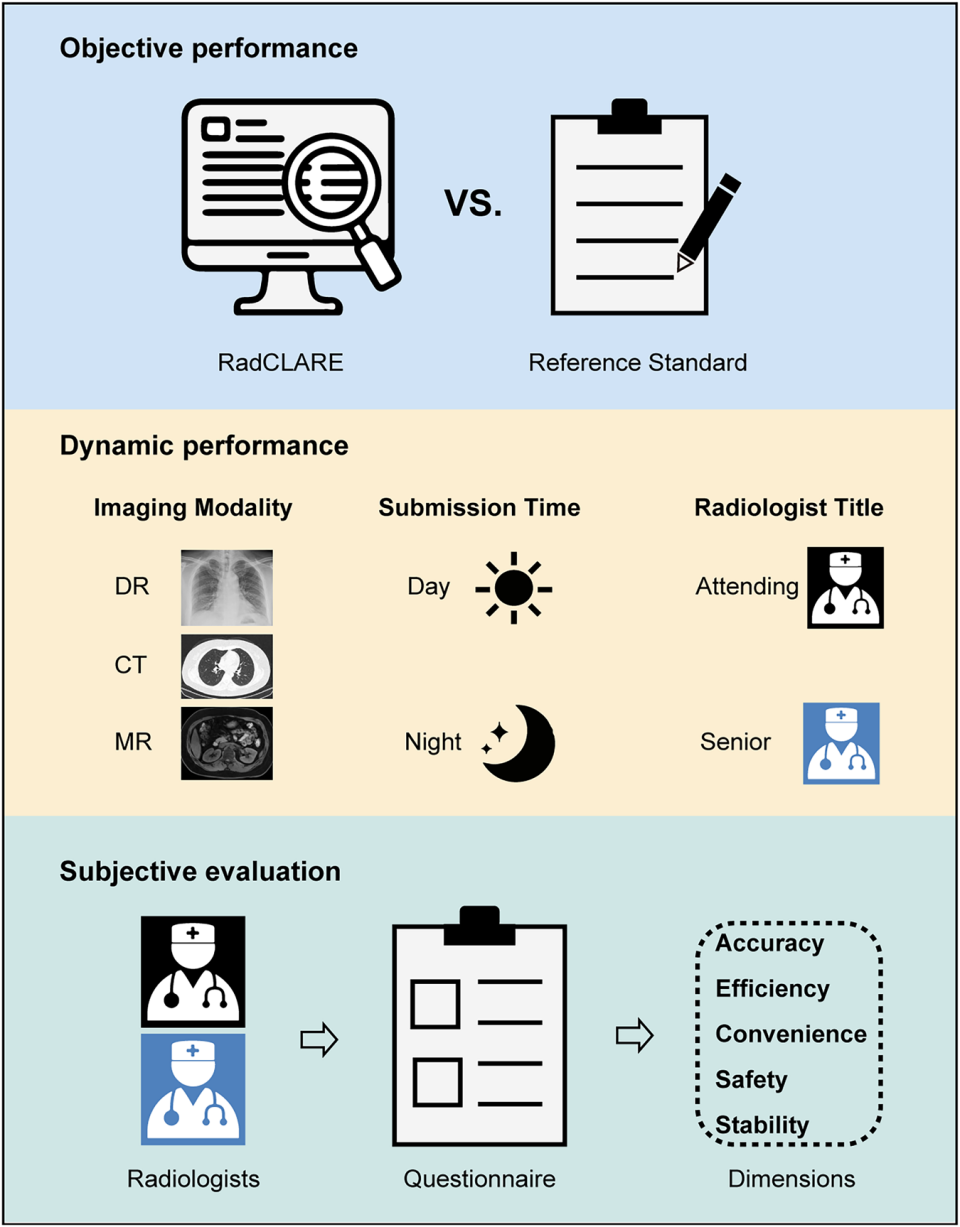
RadCLARE performance was assessed in terms of three aspects. RadCLARE, Radiology-specific clinical language anomaly recognition engine

The performance of RadCLARE was evaluated across three key aspects (Fig. 2).

#### Objective performance

To assess the accuracy of RadCLARE in error detection, 1,000 finalized reports were selected from the BTD. Two experienced radiologists (Q.F. and F.P., with 15 and 12 years of experience, respectively) independently annotated errors in these reports, using the finalized reports as the reference standard. Discrepancies between the two annotators were resolved through consensus. To ensure the accuracy of annotations, a third radiologist (L.Y., with 26 years of experience) reviewed all reports to confirm the absence of additional errors. These manually annotated reports were then processed by RadCLARE for automated error detection. We evaluated the number of correctly detected errors by RadCLARE.

#### Dynamic performance

To comprehensively evaluate the impact of RadCLARE on the quality of radiology reports before and after system implementation, we compared the error rate across different subgroups based on:

- imaging modality (CT, MRI, or DR);
- submission time (daytime: 08:00 AM–5:59 PM; nighttime: 06:00 AM–07:59 AM);
- radiologist title (attending or senior physician).

These analyses provided insights into RadCLARE’s role in reducing errors across different reporting conditions.

#### Subjective evaluation

A structured questionnaire was administered to both senior and attending radiologists to assess RadCLARE’s usability in clinical practice. Participants provided feedback on four single-choice questions and rated the system (0-to-5 points) across five dimensions: accuracy, efficiency, convenience, safety, and stability. These subjective assessments complemented the objective performance analyses, offering a comprehensive evaluation of RadCLARE’s clinical utility.

### Statistical analysis

All analyses were performed using R software (version 4.3.1) and MedCalc Statistical Software (version 23.0.1). The error rates of radiology reports before and after RadCLARE’s implementation were compared using the χ² test. Model performance was assessed by accuracy, precision, recall, and F1-score. The 95% confidence intervals (CIs) were estimated with nonparametric bootstrapping with 1,000 resamples. To identify factors associated with changes in the error rate, multiple linear regression models were applied, incorporating variables such as the total number of reports, imaging modality, reporting time, and titles of radiologists. Cohen's κ coefficient was used to evaluate the inter-reader agreement in error detection, and Gwet AC1 was used to assess inter-reader reliability for error classification. A radar plot was used to show mean scores of the model across five dimensions, and the Wilcoxon rank-sum test was used to compare the scores given by the attending and senior doctors. All statistical tests were two-tailed, with *p*-values < 0.05 considered statistically significant.

## Results

### Basic characteristics

During the test phase, a total of 30 attending radiologists and 16 senior radiologists participated in the study. The BTD comprised 571,264 finalized reports, while the ETD included 874,230 finalized reports. The baseline characteristics of the two datasets are summarized in Table 1.

**Table 1:** Basic characteristics

Reports	Overall	BTD	ETD
Total number of reports	1,444,294	571,264	873,030
Imaging modality			
DR	413,095 (28.6%)	151,819 (26.6%)	261,276 (29.9%)
CT	772,779 (53.5%)	318,255 (55.7%)	454,524 (52.1%)
MRI	258,420 (17.9%)	101,190 (17.7%)	157,230 (18.0%)
Time of submission			
Daytime	1,240,641 (85.9%)	489,733 (85.7%)	750,908 (86.0%)
Nighttime	203,653 (14.1%)	81,531 (14.3%)	122,122 (14.0%)
Title of radiologist			
Attendings	1,006,905 (69.7%)	396,157 (69.3%)	610,748 (70.0%)
Seniors	437,389 (30.3%)	175,107 (30.7%)	262,282 (30.0%)

*BTD* Baseline test dataset, *ETD* Experimental test dataset, *CT* Computed tomography, *DR* Digital radiography, *MRI* Magnetic resonance imaging

### Objective performance

Among the 1,000 annotated reports from the BTD, a total of 506 errors were identified as the reference standard. The two radiologists demonstrated good inter-reader agreement in error detection, with a Cohen κ coefficient of 0.77 (95% CI: 0.73–0.81). For errors mutually identified by both radiologists, the agreement on error classification exhibited excellent reliability, with a Gwet AC1 of 0.94 (95% CI: 0.90–0.96). RadCLARE successfully detected 437 of these 506 errors, yielding a recall of 86.4% (95% CI: 83.2–89.3). However, the system incorrectly flagged 58 reports as erroneous when no errors were present. Overall, RadCLARE demonstrated great performance in error detection with a precision of 88.3% (95% CI: 85.3–90.9), an accuracy of 87.3% (95% CI: 85.2–89.2), and an F1-score of 87.4% (95% CI: 85.0–89.3). The detection rates for the different error types in radiology reports are listed in Table 2.

**Table 2:** Detection rates for different error types in radiology reports by RadCLARE

Error types	Detection rate (%)
Overall	86.4 (437/506)
Spelling mistakes	88.1 (273/310)
Side confusion	77.1 (27/35)
Incorrect measurement units	85.0 (34/40)
Gender errors	87.5 (21/24)
Age errors	88.9 (16/18)
Mismatched imaging modalities	84.4 (38/45)
Other	82.4 (28/34)

### Changes in error rates of radiology reports

**Fig. 3:**
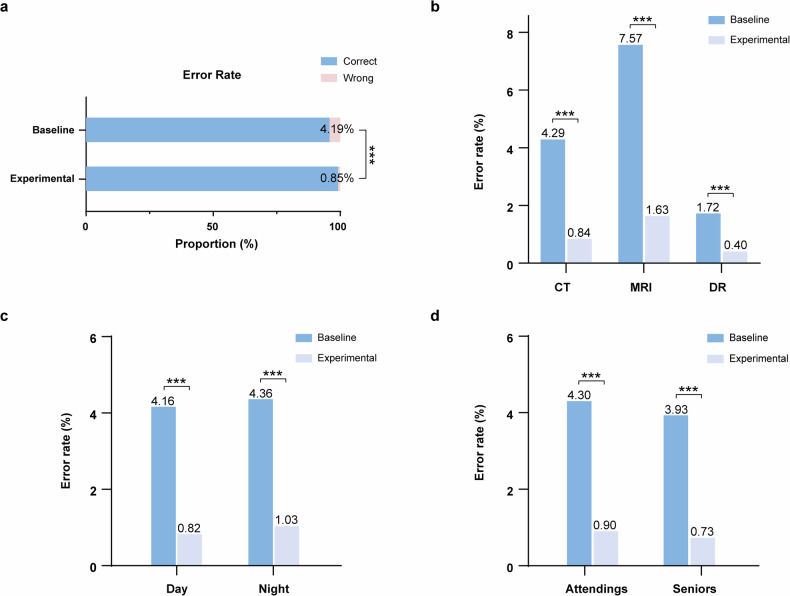
Bar graphs showed the changes in the semantic error rate of finalized reports before (Baseline) and after (Experimental) the introduction of RadCLARE. **a** Overall; **b** different imaging modality; **c** different submission time; and (**d**) different radiologist title. RadCLARE, Radiology-specific clinical language anomaly recognition engine

In the overall analysis, radiology reports with RadCLARE assistance had a lower semantic error rate compared with those without RadCLARE assistance (error rate, 0.85% [7,408 of 873,030] *versus* 4.19% [23,909 of 571,264]; *p* < 0.001) (Fig. 3a). In the subgroup analysis of different imaging modalities, the results showed that semantic errors in CT (error rate, 0.84% [3,796 of 454,524] *versus* 4.29% [13,641 of 318,255]; *p* < 0.001), MRI (error rate, 1.63% [2,558 of 157,230] *versus* 7.57% [7,656 of 101,190]; *p* < 0.001) and DR (error rate, 0.40% [1,054 of 261,276] *versus* 1.72% [2,612 of 151,819]; *p* < 0.001) reports were significantly reduced after the application of RadCLARE (Fig. 3b). Similar trends were observed across subgroups stratified by report submission time and radiologist title (Fig. 3c, d). Regarding the specific error categories, error rates for various error types were significantly lower after the introduction of RadCLARE compared to the BTD (Table 3).

**Table 3:** Comparison of error rates for different error types in radiology reports

Error types	Error rate (%)		*p*-value
	BTD	ETD	
Spelling mistakes	4.13 (23,573/571,264)	0.87 (7,614/873,030)	< 0.001
Side confusion	0.06 (363/571,264)	0.02 (212/873,030)	< 0.001
Incorrect measurement units	0.02 (117/571,264)	0.01 (50/873,030)	< 0.001
Gender errors	0.04 (231/571,264)	0.01 (109/873,030)	< 0.001
Age errors	0.04 (210/571,264)	0.02 (198/873,030)	< 0.001
Mismatched imaging modalities	0.18 (1,025/571,264)	0.09 (787/873,030)	< 0.001
Other	0.19 (1,107/571,264)	0.10 (898/873,030)	< 0.001

Line graphs were plotted to reveal changes in the semantic error rate per month for different subgroups (Supplementary Fig. S2). Multiple linear regression analysis showed that the use of RadCLARE (β = -1.035; *p* < 0.001), total number of reports (β = 0.107; *p* < 0.001), and proportion of MRI reports (β = 0.030; *p* = 0.033) were significantly associated with the semantic error rate (Table 4).

**Table 4:** Multiple linear regression model predicting changes in error rate per month

Parameter	β	Standard error	*p*-value
RadCLARE			
No	Reference		
Yes	-1.035	0.095	< 0.001
Total number of reports	0.107	0.022	< 0.001
Proportion of MRI reports	0.030	0.014	0.033
Proportion of nighttime reports	0.016	0.016	0.319
Proportion of reports from seniors	-0.007	0.014	0.623

*MRI* Magnetic resonance imaging, *RadCLARE* Radiology-specific clinical language anomaly recognition engine

#### Subjective evaluation

**Fig. 4:**
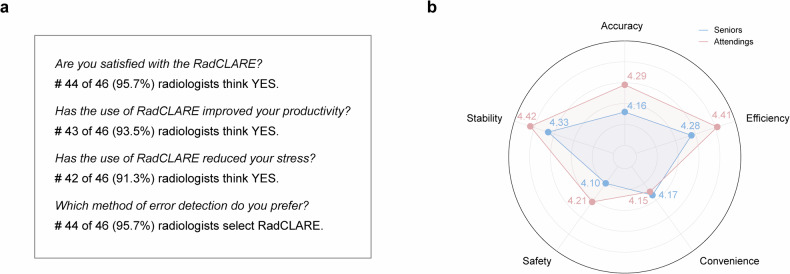
**a** Detailed statistics of four single-choice questions, (**b**) Radar chart presenting the mean scores of RadCLARE across five dimensions. RadCLARE, Radiology-specific clinical language anomaly recognition engine

To assess the subjective impressions about RadCLARE, questionnaires were sent to the involved 30 attending radiologists and 16 senior radiologists. Of 46 participants, 44 (95.7%) expressed satisfaction with RadCLARE. Furthermore, 43 (93.5%) agreed that RadCLARE improved their productivity, 42 (91.3%) felt that it reduced stress, and 44 (95.7%) preferred using RadCLARE for detecting errors in reports (Fig. 4a). The radar map shows mean scores of RadCLARE across five dimensions (Fig. 4b). Senior and attending radiologists gave similar ratings to RadCLARE in terms of accuracy (4.16 ± 0.30 [mean ± standard deviation] *versus* 4.29 ± 0.30, *p* = 0.118), efficiency (4.28 ± 0.28 *versus* 4.41 ± 0.27, *p* = 0.100), convenience (4.17 ± 0.28 *versus* 4.15 ± 0.28, *p* = 0.843), safety (4.10 ± 0.30 *versus* 4.21 ± 0.20, *p* = 0.290), and stability (4.33 ± 0.26 *versus* 4.42 ± 0.29, *p* = 0.156) (Supplementary Table S6).

## Discussion

Accurate and timely radiology reports are essential for clinical diagnosis and treatment. However, errors and discrepancies in radiology reports may occur due to heavy workload, poor image quality, and external interruptions [18, 19]. To address these challenges, we introduced RadCLARE, the first tool designed for error correction and quality control of radiology reports in China. Our study clearly demonstrates the remarkable effectiveness of RadCLARE in reducing errors in radiology reports. After the introduction of RadCLARE, the semantic error rate of finalized reports decreased substantially from 4.19% to 0.85%. This finding suggests that integrating domain-specific knowledge into LLMs could lead to meaningful performance gains in real clinical settings.

Semantic errors in radiology reports, including spelling, location, and gender errors, can affect the credibility of the radiologist or lead to inappropriate treatments with potentially serious consequences for patients [20]. In our study, RadCLARE detected semantic errors in real time and promptly delivered targeted suggestions to the responsible radiologist, which may have contributed to the observed reduction in error rates. Similar to the findings of Lumish et al [21], several radiologists told us that their increased awareness of these errors led them to avoid them more carefully, suggesting that RadCLARE’s reminders had a positive impact on doctors. RadCLARE enhanced the reporting physicians’ understanding of errors and thus improved their reporting skills, which is another clinically significant outcome of our system [22].

Using manual error detection as the reference standard, we found that RadCLARE performed well in detecting semantic errors (precision, 88.3%; recall, 86.4%; F1-score, 87.4%). This highlights the strength of LLMs in capturing semantic nuances and improving the consistency of radiology reports. Notably, the reference standard was established with high inter-reader agreement, further supporting the reliability of the evaluation results. Schmidt et al [7] compared the performances of five generative LLMs to detect speech recognition errors in radiology reports and found that the GPT-4 demonstrated high accuracy in detecting clinically significant errors (precision, 76.9%; recall, 100%; F1 score, 86.9%) and not clinically significant errors (precision, 93.9%; recall, 94.7%; F1 score, 94.3%). Another study used GPT-4 to detect 150 errors (including omission, insertion, spelling, side confusion, and others) in 200 radiology reports, with a GPT-4 detection rate of 82.7% [8].

Compared with general LLMs such as GPT-4, RadCLARE has several advantages. First, its higher precision in semantic error detection is largely attributable to domain-specific training on a large-scale corpus comprising medical imaging knowledge bases, radiology reports, and homophone dictionaries. Second, RadCLARE integrates explicit knowledge rules into the model inference pipeline, enhancing its ability to capture radiology-specific semantic inconsistencies. Third, although RadCLARE is seamlessly integrated into the current radiology workflow, it obtains data through the secure, encrypted server and implements data protection measures to ensure the privacy and security of the data. Finally, it does not directly modify the content of radiology reports, which ensures the security of the operational process. While AI tools can enhance clinical efficiency, radiologist oversight remains essential to ensure accuracy [23, 24].

Our study has several limitations. First, this study is currently limited to a single-center dataset in Chinese, which may affect the generalizability of the findings. In the future, we plan to conduct relevant randomized controlled trials to further evaluate the utility of RadCLARE in radiology practice. Second, although the radiologists participating in the subjective evaluation were unaware that the system was developed and deployed by the local institution, the lack of a rigorous blinding design means that the risk of positive bias in their subjective feedback cannot be fully excluded. Third, our team does not have the resources to manually review all reports due to the sheer volume of reports at our institution, which means that a small number of errors in radiology reports may be missed. Like other LLMs, RadCLARE may occasionally produce hallucinations. To address this issue, we have implemented a dynamic hot-fix mechanism that enables the model to learn from feedback and automatically avoid similar errors. Finally, our study focused on seven categories of semantic errors and did not include diagnostic errors associated with images. The applicability of RadCLARE could be improved by expanding the error categories in the future. In addition, given the rapid pace of LLM development, future versions of RadCLARE may explore integration with newer-generation models to assess whether these advances can further enhance semantic error detection performance.

Notably, the current error detection mechanism of RadCLARE is specifically designed for the linguistic characteristics of Chinese radiology reports. To our knowledge, this is the first study to introduce and systematically evaluate an error correction and quality control tool for Chinese radiology reports in a real clinical setting. However, there will be a series of challenges to scaling up the system to a multilingual clinical environment. Firstly, Chinese reports have significant linguistic characteristics regarding grammatical structure, expression methods, and terminology standards, which may limit the direct applicability of RadCLARE to other languages. Second, common spelling errors involving homophones and near-homophones are language-specific phenomena in Chinese and are less prevalent in other languages. In contrast, errors such as incorrect measurement units or gender errors are language-independent and provide a foundation for cross-lingual adaptation. Despite these challenges, extending RadCLARE to multilingual contexts remains feasible. On one hand, the underlying language model on which RadCLARE is built demonstrates strong multilingual generalization capabilities, allowing it to handle text processing tasks across different languages. On the other hand, with the integration of high-quality, multilingual annotated datasets for further training and optimization, RadCLARE can be effectively adapted to English and other languages, achieving broader cross-language applications.

In conclusion, RadCLARE, an automated clinical language engine based on BERT, significantly reduced semantic errors in Chinese radiology reports. This study highlights the critical role of integrating domain expertise and LLMs to improve the quality of radiology reports. An intelligent system such as RadCLARE is indispensable given the increasing workload and varying levels of expertise of radiologists.

## Supplementary information

**Additional file 1: Supplementary Table S1.** Comprehensive definitions and categories of errors in radiology reports. **Supplementary Table S2.** Examples of commonly used phrases from imaging reports in the high-quality corpus used for error generation. **Supplementary Table S3.** Examples of homophone/near-homophone in the high-quality corpus used for error generation. **Supplementary Table S4.** Examples of error generation. **Supplementary Table S5.** Examples of Custom Entity Relationship Database. **Supplementary Table S6.** Subjective evaluations of RadCLARE in senior and attending radiologists. **Supplementary Fig.S1.** Illustration of the RadCLARE user interface for semantic error detection. Once the report has been finalised, the system automatically initiates error detection and sends error alerts back to the front end. The left panel displays a notification showing the number of errors detected. The errors in the report are highlighted in color in the right panel, along with correction suggetions. **Supplementary Fig.S2.** Line plots showing the semantic error rate of finalized reports per month. RadCLARE was implemented in June 2023 and the error rate of all groups decreased significantly. (a) Overall; (b) Different imaging modality; (c) Different time period for submission of the report (day: 8:00 AM to 5:59 PM; night: 6:00 AM to 7:59AM); (d) Different physician's title.

## Data Availability

The datasets and source code used in this study are not currently publicly available due to intellectual property considerations. Data and code can be provided upon reasonable request to the corresponding author.

## Abbreviations

AI: Artificial intelligence
BERT: Bidirectional encoder representations from transformers
BTD: Baseline test dataset
CI: Confidence interval
CT: Computed tomography
DR: Digital radiography
ETD: Experimental test dataset
LLMs: Large language models
MRI: Magnetic resonance imaging
RadCLARE: Radiology-specific clinical language anomaly recognition engine
