# Supplementary material for: RadCLARE: an automated clinical language engine for detecting semantic errors in radiology reports
Source: Eur Radiol Exp. 2025 Dec 22;9:120. doi: 10.1186/s41747-025-00659-x (PMC12722592; doi:10.1186/s41747-025-00659-x)
Supplement: Supplementary file 1 — Additional file 1: Supplementary Table S1. Comprehensive definitions and categories of errors in radiology reports. Supplementary Table S2. Examples of commonly used phrases from imaging reports in the high-quality corpus used for error generation. Supplementary Table S3. Examples of homophone/near-homophone in the high-quality corpus used for error generation. Supplementary Table S4. Examples of error generation. Supplementary Table S5. Examples of Custom Entity Relationship Database. Supplementary Table S6. Subjective evaluations of RadCLARE in senior and attending radiologists. Supplementary Fig.S1. Illustration of the RadCLARE user interface for semantic error detection. Once the report has been finalised, the system automatically initiates error detection and sends error alerts back to the front end. The left panel displays a notification showing the number of errors detected. The errors in the report are highlighted in color in the right panel, along with correction suggetions. Supplementary Fig.S2. Line plots showing the semantic error rate of finalized reports per month. RadCLARE was implemented in June 2023 and the error rate of all groups decreased significantly. (a) Overall; (b) Different imaging modality; (c) Different time period for submission of the report (day: 8:00 AM to 5:59 PM; night: 6:00 AM to 7:59AM); (d) Different physician's title. [file 41747_2025_659_MOESM1_ESM.pdf]

RadCLARE: an automated clinical language engine for detecting semantic errors in radiology reports

ELECTRONIC SUPPLEMENTARY MATERIAL

Supplementary Table S1 Comprehensive definitions and categories of errors in radiology reports

Error category	Definition	Examples
Spelling mistakes	The omission of relevant words, inappropriate words, incorrect word substitutions, insertions or word confusions	“结节” (nodule) is written as “姐姐” (sister)*.
Side confusion	Inconsistency in orientation between the findings and impressions sections	The impression section showed that the lesion was located in the “left lung”, but the finding section showed it was in the “right lung”.
Incorrect measurement units	Mistakes in measurement units	“mm” is written as “cm”.
Gender errors	Inconsistency between the description in the report and the sex of the examiner	The report mentions “a prostate gland” in a female patient.
Age errors	Inconsistency between the description in the report and the age of the examiner	The report mentions “senile degeneration” in a 9-year-old patient.
Mismatched imaging modalities	Inconsistency between the description in the report and the imaging modality	The CT report describes the lesion as “low signal”.
Other	Unclassified in above types	Incorrect date entries, template errors, punctuation mistakes and so on.

Note: *In Chinese, “结节” (nodule) and “姐姐” (sister) are pronounced similarly, so it is easy to make mistakes when entering them in pinyin (the standard system of romanized spelling for transliterating Chinese).

Supplementary Table S2 Examples of commonly used phrases from imaging reports in the high-quality corpus used for error generation

Types	Commonly used phrases
Chest CT description	胸廓对称 (thorax symmetrical)
Chest CT description	纹理清晰 (texture clear)
Chest CT description	未见 (not seen)
Chest CT impression	多考虑为 (more likely considered as)
Chest CT impression	诊断可能 (diagnosis possible)
Chest CT impression	对比前片 (compared with previous film)
Brain MRI description	左右对称 (left-right symmetry)
Brain MRI description	中线结构居中 (midline structures centered)
Brain MRI description	异常信号 (abnormal signal)
Brain MRI impression	未见异常 (no abnormalities detected)
Brain MRI impression	脑萎缩 (brain atrophy)
Brain MRI impression	缺血性病变 (ischemic lesion)
Abdominal CT description	肠管扩张 (bowel dilatation)
Abdominal CT description	气液平面 (gas-liquid level)
Abdominal CT description	胃壁增厚 (gastric wall thickening)
Abdominal CT impression	腹腔积液 (abdominal fluid)
Abdominal CT impression	小肠梗阻 (small bowel obstruction)
Abdominal CT impression	急性阑尾炎 (acute appendicitis)
Spinal MRI description	生理曲度 (physiological curvature)
Spinal MRI description	骨质增生 (hyperosteogeny)
Spinal MRI description	硬膜囊受压 (the compression of the dural sac)
Spinal MRI impression	腰椎退行性变 (lumbar spinal degeneration)
Spinal MRI impression	压缩性骨折 (compression fracture)
Spinal MRI impression	椎管狭窄 (spinal stenosis)

CT Computed tomography, *MRI* Magnetic resonance imaging.

Supplementary Table S3. Examples of homophone/near-homophone in the high-quality corpus used for error generation

Specialized vocabulary	Homophone/near-homophone
未见(not seen)	违建(illegal construction), 未检(not detected), 危机(crisis)
表面(surface)	表明(show), 表妹(cousin), 标明(mark)
结节(nodule)	姐姐(sister), 结界(boundary), 阶级(class)
左侧(left side)	坐厕(toilet), 做错(do wrong), 做出(make)
走行(course)	走形(out of shape), 走向(go), 走秀(show)
纵隔(mediastinum)	纵膈(mediastinum), 总格(zong ge), 总共(total)
心包(pericardium)	信报(newspaper), 新包(new package), 新版(new version)
肠管(bowel)	场馆(venue), 长骨(long bone), 长管(long tube)
畸形(malformation)	急性(acute), 记性(memory), 机型(machine)
内镜(endoscopy)	内径(inner diameter), 内景(interior), 内经(Yellow Emperor)

Supplementary Table S4. Examples of error generation

Original report	Types of error generation	New errored report
胸部正位片未见异常，必要时进一步检查。<br>Chest radiograph showed no abnormality and further examinations could be performed if necessary.	Homophone alternation	胸部正位片违建异常，必要时进一步检查。<br>Chest radiograph illegal construction no abnormality and further examinations could be performed if necessary.
胸部 CT 显示双肺多发小结节，建议复查。<br>Chest CT showed multiple small nodules in both lungs and re-examination was recommended.	Homophone alternation	胸部 CT 显示双肺多发小结界，建议复查。<br>Chest CT showed multiple small boundaries in both lungs and re-examination was recommended.
双肺纹理增多，透亮度增加。<br>The texture of both lungs was increased and the transparency was increased.	Word omitting	双肺纹理增，透亮度增加。<br>The texture of both lungs was increased and the transparency was increased.
膀胱充盈不佳，壁光滑，内未见异常密度影。<br>The bladder was inadequately filled, the wall is smooth and no abnormal density shadows are visible inside.	Word omitting	膀胱充盈不，壁光滑，内未见异常密度影。<br>The bladder was not full, the wall is smooth and no abnormal density shadows are visible inside.
胰腺形态及密度未见明显异常，未见腹膜后淋巴结肿大。<br>There were no obvious abnormalities in the morphology and density of pancreatic and no enlargement was observed in the retroperitoneal lymph node.	Word adding	胰腺形态及密度未见明显异常异常，未见腹膜后淋巴结肿大。<br>There were no obvious abnormalities abnormalities in the morphology and density of pancreatic and no enlargement was observed in the retroperitoneal lymph node.
肝左叶见圆形低密度影，未见明显强化。<br>There was a circular low-density shadow, but no obvious enhancement was found.	Word adding	肝左叶见圆形低密度影，未见明显明显强化。<br>There was a circular low-density shadow, but no obvious obvious enhancement was found.
肝旁见一小结节，长径约 1.4cm。<br>A small nodule was seen adjacent to the liver, which was about 1.4 cm in length.	Word omitting	肝旁见一小结节，长径约 1.4 m。<br>A small nodule was seen adjacent to the liver, which was about 1.4 m in length.
右肺上叶前段见一磨玻璃结节，大小约 20×15mm。<br>There was a ground-glass nodule in the anterior segment of the superior lobe of the right lung and it was approximately 20 × 15 mm in size.	Near-homophone alternation	右肺上叶前段见一磨玻璃结节，大小约 20×15cm。<br>There was a ground-glass nodule in the anterior segment of the superior lobe of the right lung and it was approximately 20 × 15 cm in size.

Supplementary Table S5. Examples of Custom Entity Relationship Database.

Entity-1	Entity-2	Entity-3	…	Relationship
Gender-related term: 女 (female)	Anatomical location: 前列腺 (prostate)	-	-	Contradicted
Gender-related term: 男 (male)	Anatomical location: 子宫 (uterus)	-	-	Contradicted
Device-related term: 磁共振 (MRI)	Device-related term: 密度 (signal)	-	-	Contradicted
Device-related term: 磁共振 (MRI)	Device-related term: 信号 (density)	-	-	Consistent
Age-related term: 7 岁 (7 years of age)	Diagnosis: 退行性变 (degeneration)	-	-	Contradicted
Anatomical location: 肺纹理 (lung texture)	Negative sign: 清晰 (clear)	Diagnosis: 肺炎 (pneumonia)	-	Contradicted
Anatomical location: 肺纹理 (lung texture)	Positive sign: 阴影 (shadow)	Diagnosis: 肺炎 (pneumonia)	-	Consistent
Anatomical location: 心脏 (heart)	Positive sign: 增大 (enlarged)	Diagnosis: 心脏增大 (cardiomegaly)	-	Consistent
Anatomical location: 心包 (pericardium)	Positive sign: 积液 (effusion)	Diagnosis: 心包积液 (pericardial effusion)	-	Consistent
Anatomical location: 肺部 (lung)	Positive sign: 结节 (nodule)	Measurement unit: 米 (m)	-	Contradicted
Anatomical location: 肺部 (lung)	Positive sign: 结节 (nodule)	Measurement unit: 毫米 (mm)	-	Consistent

Supplementary Table S6. Subjective valuations of RadCLARE in senior and attending radiologists

Dimensions	Senior radiologists	Attending radiologists	*p*-value
Accuracy	4.16 ± 0.30	4.29 ± 0.30	0.118
Efficiency	4.28 ± 0.28	4.41 ± 0.27	0.100
Convenience	4.17 ± 0.28	4.15 ± 0.28	0.843
Safety	4.10 ± 0.30	4.21 ± 0.20	0.290
Stability	4.33 ± 0.26	4.42 ± 0.29	0.156

Supplementary Figures

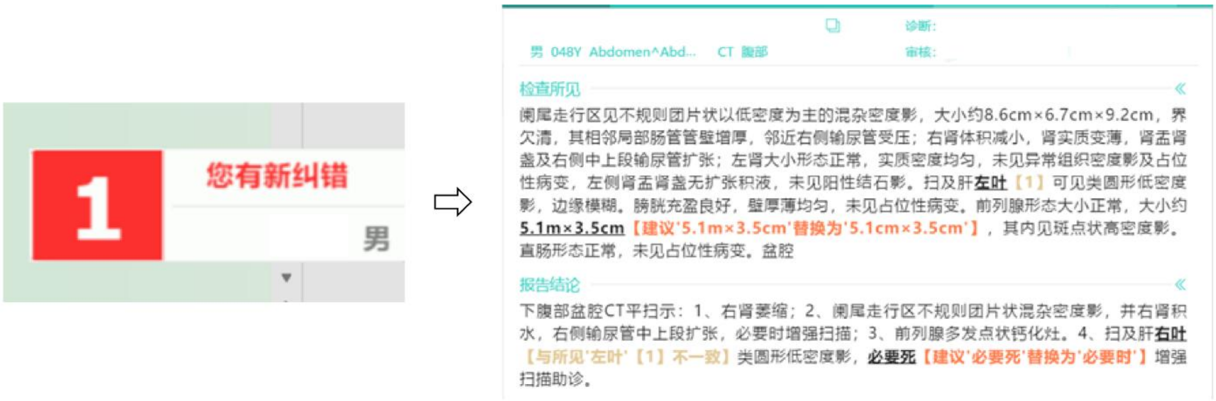

Supplementary Fig.S1 Illustration of the RadCLARE user interface for semantic error detection. Once the report has been finalised, the system automatically initiates error detection and and sends error alerts back to the front end. The left panel displays a notification showing the number of errors detected. The errors in the report are highlighted in color in the right panel, along with correction suggestions.

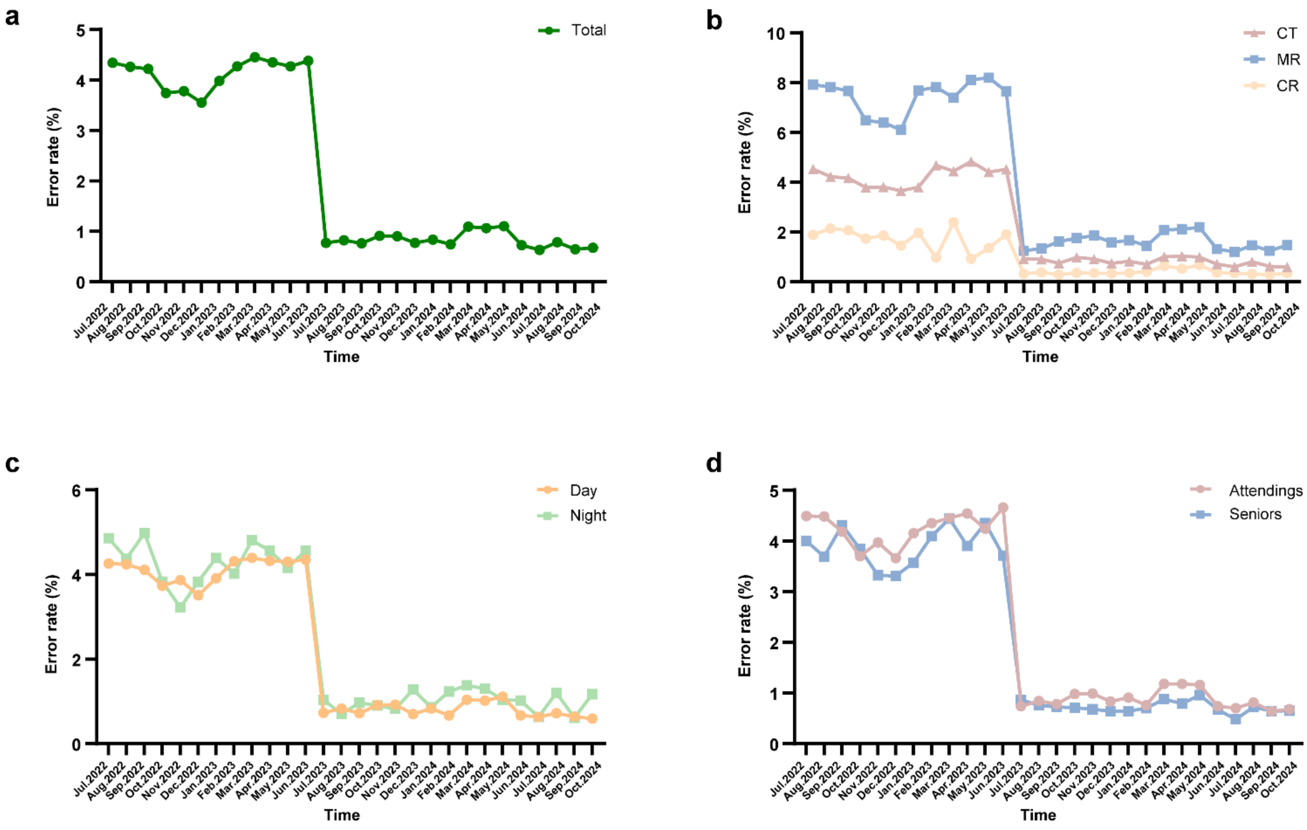

Supplementary Fig.S2 Line plots showing the semantic error rate of finalized reports per month. RadCLARE was implemented in June 2023 and the error rate of all groups decreased significantly.(a) Overall; (b) Different imaging modality; (c) Different time period for submission of the report (day: 8:00 AM to 5:59 PM; night: 6:00 AM to 7:59AM); (d) Different physician's title.
